# Provincial inequality of China’s progress towards universal health coverage: An empirical analysis in 2016–21

**DOI:** 10.7189/jogh.14.04122

**Published:** 2024-06-28

**Authors:** Yining Wang, Ruixin Wang, Mingzhu Jiang, Xiaohua Ying

**Affiliations:** 1 School of Public Health, Fudan University, Shanghai, China; 2 Key Laboratory of Health Technology Assessment (Fudan University), Ministry of Health, Shanghai, China

## Abstract

**Background:**

Achieving universal health coverage (UHC) is a crucial target shared by the Sustainable Development Goals (SDGs). As UHC levels are influenced by factors such as the regional economy and resource allocation, subnational evidence in China is urgently needed. This study aimed to monitor provincial progress from 2016 to 2021, thereby informing the development of region-specific strategies.

**Methods:**

Based on the UHC monitoring framework proposed by the World Health Organization, a UHC index was constructed comprising the service coverage dimension (16 indicators) and financial protection dimension (four indicators). In this observational study, routinely collected health data from 25 provinces (autonomous regions and municipalities) in mainland China were obtained from statistical yearbooks, relevant literature, and nationally representative surveys. The indices were calculated using geometric means. Socioeconomic inequalities among provinces were quantified using the slope index of inequality (SII) and relative index of inequality (RII).

**Results:**

From 2016 to 2021, China made laudable progress towards achieving UHC, with the index rising from 56.94 in 2016 to 63.03 in 2021. Most provinces demonstrated better performance in service coverage. Western provinces generally presented faster rates of progress, which were attributed to more substantial increases in financial protection. Despite significant disparities, with the UHC index ranging from 77.94 in Shanghai to 54.61 in Fujian in 2021, the overall equity of UHC has improved across the 25 provinces. SII decreased from 17.78 (95% confidence interval (CI) = 11.64, 23.93) to 12.25 (95% CI = 5.86, 18.63) and RII from 1.38 (95% CI = 1.29, 1.46) to 1.22 (95% CI = 1.16, 1.29). However, the non-communicable disease (NCD) domain experienced a drop in both index score and equity, underscoring the need for prioritised attention.

**Conclusions:**

In the context of SDGs and the ‘Healthy China 2030’ initiative, China has made commendable progress towards UHC, and inter-provincial equity has improved. However, substantial differences persisted. The equitable realisation of UHC necessitates prioritising the enhancement of service capacity and financial protection in less developed regions, particularly by addressing shortages in the general practitioner workforce and mitigating catastrophic payments. Developed regions should focus on preventing NCDs through effective interventions targeting key risk factors. This study provides insights for other countries to adopt comprehensive monitoring frameworks, identify subnational disparities, and introduce targeted policy initiatives.

Since the establishment of the United Nations (UN) Sustainable Development Goals (SDGs) in 2015, universal health coverage (UHC) has become a global policy priority. SDG 3.8 advocates the equitable and progressive realisation of UHC, ensuring that all individuals have access to essential health care services without suffering undue financial hardship [1]. This objective encompasses two interrelated dimensions – coverage of essential health services (SDG 3.8.1) and financial risk protection (SDG 3.8.2) [2]. SDG 3.8.1 covers the full spectrum of services for health promotion, prevention, treatment, rehabilitation, and palliative care tailored to individual needs. SDG 3.8.2, conversely, aims to avoid catastrophic payments due to illness. With the World Health Organization (WHO) establishing targets of at least 80% service coverage and 100% financial coverage by 2030 for each country, the principle of equity is at the heart of UHC endeavours [3]. This principle includes achieving better performance in low- and middle-income countries and eliminating inequities at the regional, national and sub-national levels by prioritising the needs of the most vulnerable populations [4].

Global reports and academic literature have discussed tracking progress towards UHC. The WHO and World Bank proposed a monitoring framework comprising 16 tracer indicators in the service coverage dimension and one indicator in the financial protection dimension [5]. The most recent monitoring report based on this framework has been updated in 2021 [6]. Several studies have also presented cross-country or country-specific analyses [7–10]. These studies developed and adapted effective measurement frameworks with different tracer indicators to examine current UHC levels, temporal trends, and associated correlates. Among them, several studies have used the concentration index and logistic regression models to quantify inequalities based on gender, income, and urban-rural areas, with few exploring regional variations within the country. The slope and relative indices of inequality, another widely used method for health equity research, have been adopted in some studies focusing on subnational differences in service coverage but are rarely used in comprehensive assessments of overall UHC levels [11–13].

China has consistently committed to achieving UHC objectives by incorporating UHC targets into its broad health reforms [14]. The large-scale and complex health system reform in 2009 began with increasing financial investment to expand social health insurance coverage [15]. Subsequently, considerable fiscal resources were allocated to reform the fragmented health care delivery system by enhancing primary care facilities and expanding the national essential public health programme, which provides free services to all [16]. Furthermore, health poverty alleviation, an integral component of targeted poverty alleviation initiated in 2015, offered health care services to low-income groups at nearly no cost, effectively relieving individuals’ financial burden [17,18]. UHC has also been a critical element of the ‘Healthy China 2030’ initiative, the guiding principle introduced in 2016 to steer health system reform and actions towards 2030. In 2017, the people-centred integrated care programme was launched to promote coordinated health care delivery, improve quality of care, and reduce costs [19]. In 2019, the national volume-based procurement policy was implemented to mitigate drug costs [20]. In this context, China has experienced the most significant improvements in UHC indicators in the Western Pacific Region in recent years [6]. However, challenges remain in preventing non-communicable diseases (NCDs) and controlling medical expenditure.

Previous studies have investigated the temporal trend of UHC at the national level in China. However, global studies indicate that subnational inequalities pose severe challenges in Myanmar, Ethiopia, and Iraq [21–23]. Significant variations in health policies, epidemic profiles, and economic developments have affected the advancement of provinces towards achieving UHC in China. In 2021, Eastern China had over 608 million individuals, while Central and Western China had approximately 419 and 383 million, respectively. The income per capita was CNY 110 062 (USD17 059), CNY 64 715 (USD 10 031), and CNY 62 999 (USD 9765), respectively [24]. In diverse countries, such as China, national assessments mask substantial within-country differences, impeding the equitable progress of UHC. Additionally, subnational analyses are crucial for identifying inequalities, assessing the effectiveness of policies, and formulating targeted strategies [13,25]. Despite its importance, studies evaluating the conditions in each province are relatively scarce. Except for two studies, all work has been focused solely on one dimension of UHC, or each dimension has been analysed in isolation [11,26,27]. The remaining two studies simultaneously examined service coverage and financial protection. However, both studies used cross-sectional data without performing a detailed trend analysis or quantifying socioeconomic inequality among provinces [28,29].

This study builds on previous successes in tracking progress towards UHC across provinces in China and complements them by offering a more comprehensive assessment. We first constructed a UHC index to evaluate the UHC status at the national and provincial levels from 2016 to 2021, employing an adapted monitoring framework. We then analysed socioeconomic inequality of UHC levels among provinces using the slope index of inequality (SII) and relative index of inequality (RII). This study presents methodologies for other countries to localise monitoring frameworks and comprehensively track progress towards UHC. Disaggregating data at the subnational scale facilitates the identification of challenges specific to each region and the formulation of corresponding initiatives. Furthermore, this study has policy implications for countries at comparable developmental stages to achieve UHC.

## METHODS

### Indicators and data sources

We considered the specific context of China, adapted the monitoring framework proposed by the WHO and World Bank, and ultimately incorporated 20 indicators (**Table 1**). In our terminology, UHC comprises service coverage (SC) and financial protection (FP). The 16 indicators in the SC dimension can be categorised into four domains: 1) reproductive, maternal, newborn, and child health (RMNCH), 2) infectious disease (ID), 3) NCD, and 4) service capacity and access (SCA). These indicators cover the full continuum of essential health services across the whole life course. The detailed definitions and principles underpinning our selection of tracer indicators are summarised in Appendices S1–3 in the **Online Supplementary Document**.

**Table 1:** Sample means and sources of variation in selected indicators

Indicators		SD		
	**x̄**	**Overall**	**Between-province**	**Within-province**
UHC index	59.91	7.33	7.00	2.54
SC*	77.67	4.62	3.73	2.81
RMNCH	95.81	1.78	1.44	1.08
*Maternal systematic management*	91.95	4.35	3.00	3.20
*Skilled birth attendance*	99.93	0.18	0.15	0.11
*Full immunisation*	99.28	0.70	0.56	0.43
*Child systematic management (age zero to three)*	92.47	2.77	2.40	1.44
ID	87.20	6.59	5.88	3.15
*Incidence of TB*	87.20	5.52	5.29	1.85
*Incidence of HIV/AIDS*	99.22	0.81	0.80	0.17
*Improved water source*	77.32	13.02	11.42	6.59
NCD	64.84	5.18	2.74	4.43
*Prevalence of hypertension*	73.59	5.72	5.33	2.29
*Prevalence of diabetes*	87.84	2.37	2.36	0.46
*Tobacco use*	73.90	3.60	3.08	1.95
*Alcohol consumption*	85.93	4.95	3.24	3.79
*Frequent physical exercise*	29.73	9.99	5.59	8.34
SCA	68.37	12.84	8.49	9.76
*Practising (assistant) physician density*	82.42	12.33	9.53	8.02
*GP density*	58.23	23.76	18.88	14.82
*Registered nurse density*	65.79	12.73	9.76	8.37
*Hospital bed density*	73.24	11.28	9.43	6.43
FP	46.51	9.55	9.31	2.72
*Incidence of CHE*	59.84	12.54	11.07	6.23
*UEBMI coverage*	25.06	15.57	15.74	1.76
*Effective reimbursement rate*	40.55	9.20	6.44	6.68
*OOP payment*	86.96	5.00	4.67	1.97
Observations	150			

CHE – catastrophic health expenditure, FP – financial protection, GP – general practitioner, ID – infectious disease, NCD – non-communicable disease, OOP – out-of-pocket, RMNCH – reproductive, maternal, newborn, and child health, SC – service coverage, SCA – service capacity and access, SD – standard deviation, TB – tuberculosis, UEBMI – urban employee basic medical insurance, UHC – universal health coverage, x̄ – mean

*The service coverage dimension has four domains: RMNCH, ID, NCD, and SCA.

For RMNCH, we measured maternal systematic management, skilled birth attendance, full immunisation, and child management. The IDs were monitored for pulmonary tuberculosis (TB), HIV/AIDS, and water supply. We chose the incidence rate of TB and HIV/AIDS as proxy indicators for effective coverage. Without better treatment data, such incidence-based measures were good reflections of access to quality care after an unpreventable infection and have been previously used in relevant literature [8]. NCDs were assessed considering hypertension, diabetes, and three chronic disease risk factors. We omitted cervical and breast cancer screening rates, which are important indicators for prevention services, because of data unavailability. For SCA, we tracked health worker and facility density, including physicians, general practitioners (GPs), nurses, and hospital beds. We did not use inpatient admission as an indicator because over-hospitalisation is common in China [30]. For FP, except for catastrophic health expenditure (CHE), we also incorporated health insurance coverage, effective reimbursement rate (ERR), and out-of-pocket (OOP) payment to mitigate the potentially misleading effects of a single indicator. From 2016 to 2021, routinely collected health data at the national and provincial levels were gathered from population-based household surveys and administrative data.

Further, 12 indicators were derived through routine monitoring. Indicators in the RMNCH, ID, and SCA domains were sourced primarily from the China health statistics yearbook. The two indicators in the FP dimension were calculated using data from the China Statistical Yearbook, the Health Statistics Yearbook, and the China Medical Insurance Statistical Yearbook. Water sanitation was extracted from the China Social Statistical Yearbook, while vaccination coverage was obtained from relevant literature [31,32].

The eight indicators were derived from nationally representative household surveys. The prevalence of chronic diseases was collected from the Global Burden of Diseases Study 2019, the China Hypertension Study, and the relevant literature [33–35]. The study designs of these surveys have been disclosed in the pertinent literature. The chronic disease risk factors and two financial indicators were calculated in three rounds (2016, 2018, and 2020) of the China Family Panel Study (CFPS). Appendix S2 in the **Online Supplementary Document** describes the sampling methods used for the CFPS. As the CFPS was mainly sampled from 25 provinces (excluding Hainan, Inner Mongolia, Tibet, Qinghai, Ningxia, and Xinjiang), the few samples from the other six provinces could have caused a large bias. Therefore, we concluded by incorporating 25 provinces into our study.

### Index construction

All the indicators were rescaled to values from zero to 100, with higher scores indicating better performance. The transformation and rescaling process involved four steps, with the specific details of each indicator provided in Appendix S3 in the **Online Supplementary Document**. First, the missing values were imputed using linear interpolation and extrapolation. Second, we used the complements of five negative indicators in the NCD domain to capture the proportion of people without chronic diseases or risky behaviours [6,28]. Third, some indicators in the NCD, SCA, and FP categories required transformation based on designated target values [8,9,36]. These target values were established using national policy targets or international standards [37]. Indicators that were not subjected to transformation retained their actual values. The concluding step involved multiplying all the indicators by 100, with 100 as the ideal target value.

Based on the transformed data, our overall UHC index was calculated as the geometric average of the SC and FP indices with equal weights, consistent with previous studies [7,9,10,28]. The SC index was computed as an unweighted geometric average of the scores across the four domains. These domain scores were derived from the geometric average of the rescaled values for the selected indicators. The FP index was constructed as the geometric mean of the relative values of the four indicators. We opted for the geometric mean as the calculation method because it favours equal coverage levels instead of higher coverage for some dimensions at the expense of others. This could indicate how policymakers traded off SC against FP [10]. The characteristics of the continuous indicators are presented using means and standard deviations.

### Statistical analysis

For the constructed indices, we quantified the socioeconomic inequalities using a regression-based SII and RII. The SII measures absolute inequality, whereas the RII measures relative inequality. The SII and RII were estimated by regressing each index score on the province’s category on a socioeconomic scale [38,39]. The categories were ranked by the provinces’ gross domestic product (GDP) per capita on a scale of zero to one. Each category covered a range on the scale proportional to its population size and was assigned a midpoint of the range [40]. In this case, the SII can be interpreted as the absolute difference between two hypothetical extremes, from the province with the lowest GDP per capita to the one with the highest. The RII was defined as the ratio between the highest and lowest in the socioeconomic hierarchy [11,41,42]. Therefore, an RII of one would imply no difference in relative terms, whereas an SII of zero would imply no difference in absolute terms. Tracking the magnitude of these two composite indices enables observing the marginal effects of the pertinent policies and identifying the areas in urgent need of attention [43]. We estimated SII and RII with 95% confidence intervals (CIs).

To verify the robustness of our results, we performed sensitivity analyses under different scenarios. These scenarios involved substituting alternative indicators, employing different rescaling methods, and using the arithmetic mean for the calculation. The sensitivity analysis results are presented in Appendix S7 in the **Online Supplementary Document**. All statistical analyses were performed using MS Excel (Microsoft Corporation, Seattle, Washington, USA) and R, version 4.2.1 (R Core Team, Vienna, Austria).

## RESULTS

### Overall UHC index

**Figure 1:**
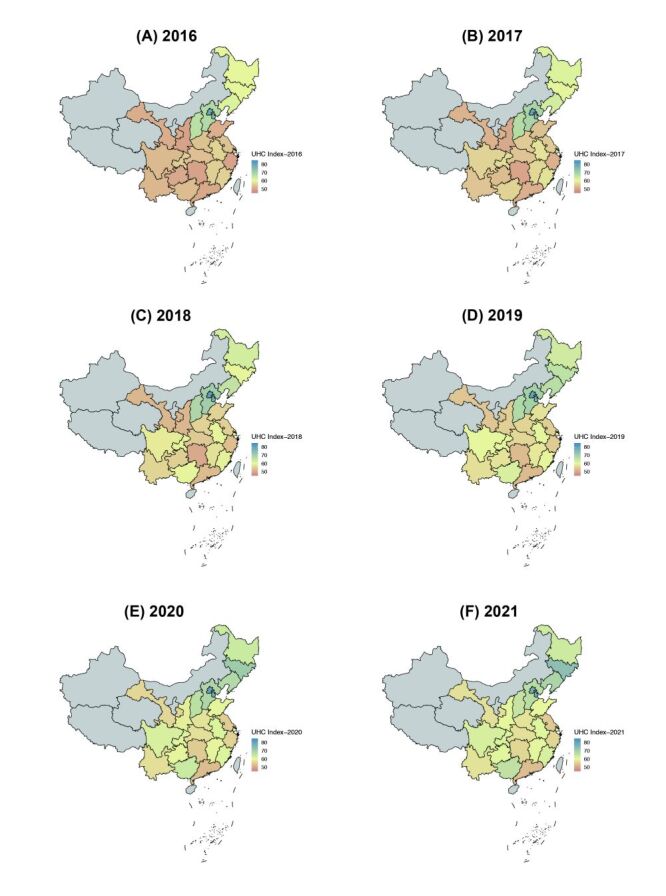
Overall UHC index scores by province across time (2016–21). **Panel A.** 2016. **Panel B.** 2017. **Panel C.** 2018. **Panel D.** 2019. **Panel E.** 2020. **Panel F.** 2021. UHC – universal health coverage

**Figure 2:**
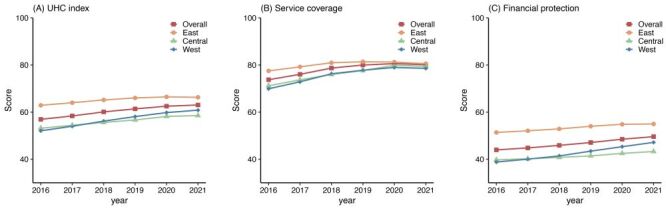
Scores of UHC index, service coverage, and financial protection by regions across time (2016–21). **Panel A.** UHC index scores. **Panel B.** Service coverage scores. **Panel C.** Financial protection scores. Overall scores represent China’s national scores. UHC – universal health coverage

We saw substantial progress in achieving UHC across most provinces; however, the pace slowed down over time (**Figure 1**, panels A–F). Based on the comprehensive monitoring framework, the UHC index in China improved from 56.94 in 2016 to 61.39 in 2019, and 63.03 in 2021. The annual improvement rate in index values was 1.26% (95% CI = 0.99, 1.53) from 2016 to 2021, slightly slower than the period between 2016–19. Provinces showed variable rates of progress. The provinces with the highest annualised rate of improvement were mainly located in the Western region (**Figure 2**, panels A–C). Tianjin (rate = 2.99%; 95% CI = 1.33, 4.64) and Chongqing (rate = 2.63%; 95% CI = 2.09, 3.18) had the fastest rates, followed by Guangxi, Gansu, and Sichuan, all exceeding 2% on average. Conversely, the smallest improvements were observed in Fujian (rate = 0.13%; 95% CI = –0.24, 0.51) and Shanghai (rate = 0.61%; 95% CI = 0.52, 0.70) in the Eastern region. The Eastern region also included the provinces that worsened in the UHC index, namely Jiangsu, Beijing, and Zhejiang. Regarding the change in progress rates, the paces slowed down in 2020–21 in most provinces, excluding Tianjin, Shanxi, Jiangxi, Guangxi, and Yunnan.

**Figure 3:**
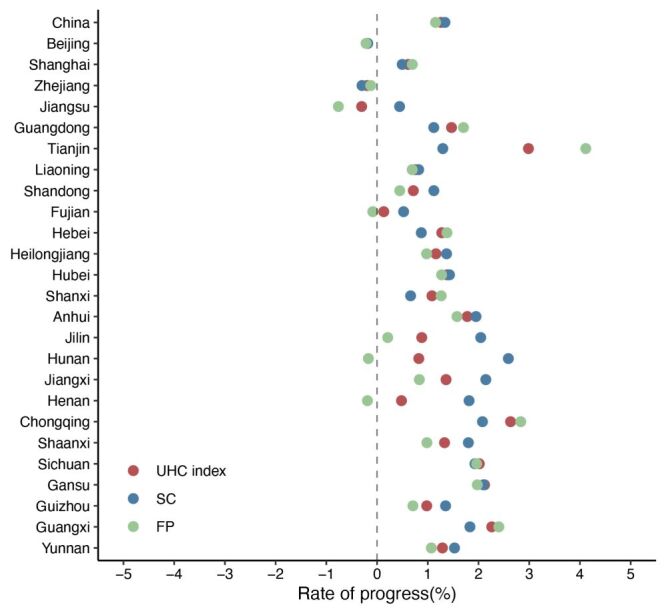
Rates of progress of UHC index, service coverage, and financial protection by province across time, presented in percentages (2016–21). The Eastern provinces include Beijing, Shanghai, Zhejiang, Jiangsu, Guangdong, Tianjin, Liaoning, Shandong, Fujian, and Hebei. Central provinces include Heilongjiang, Hubei, Shanxi, Anhui, Jilin, Hunan, Jiangxi, and Henan. Western provinces include Chongqing, Shaanxi, Sichuan, Gansu, Guizhou, Guangxi, and Yunnan. UHC – universal health coverage, SC – service coverage, FP – financial protection

While most provinces exhibited upward trends in UHC performance, the improvement patterns diverged (**Figure 3**). Consistent with the national level, an overwhelming majority of provinces progressed towards both SC and FP. However, the speed of the movement varied. Most Western provinces showed a higher rate of progress in FP than in SC. For instance, Chongqing progressed at the rate of 2.83% (95% CI = 2.77, 2.90) in FP and 2.08% (95% CI = 0.74, 3.42) in SC. This pattern has also been observed in other provinces, such as Tianjin. In contrast, the prevalent trend among the Central provinces was a faster increase in SC than in FP. For instance, Jilin had an improvement of 2.04% (95% CI = 1.23, 2.85) in SC and 0.21% (95% CI = –0.03, 0.45) in FP annually on average. Four provinces – Jiangsu, Fujian, Henan, and Hunan – saw an increase in SC index values and a decrease in F*P* values, resulting in an uncertain direction for the composite index scores. No provinces traded off SC against FP. Despite exhibiting downward trends in both dimensions, Beijing and Zhejiang experienced non-significant declines. These reductions in the two socially and economically advanced provinces were partly due to their high baseline scores. Appendices S4–5 in the **Online Supplementary Document** provide further details on each province’s progress rate with a 95% CI.

The past six years have seen reduced inequality between provinces regarding UHC, although sizeable heterogeneity remained (**Table 2**). In 2016, the SII was 17.78 (95% CI = 11.64, 23.93), decreasing to 12.25 (95% CI = 5.86, 18.63) by 2021. Similarly, the RII declined significantly from 1.38 (95% CI = 1.29, 1.46) in 2016 to 1.22 (95% CI = 1.16, 1.29) in 2021. These two summarised indices indicated smaller differences across China’s absolute and relative terms. However, considerable variations persisted among provinces’ progress towards accomplishing UHC goals. The Eastern region achieved consistently higher scores than the Western and Central regions. In 2021, the index scores ranged from 75 or higher in Shanghai (77.94) and Beijing (77.09) to lower than 55 in Henan (53.08) and Fujian (54.61). Provinces in the Western area generally clustered in the high 60s, while those in the central area lagged, clustering in the low 60s, which was well below the national average.

**Table 2:** SII and RII for overall UHC index across time (2016–21)

Year	National score	Highest score by province	Lowest score by province	SII (95% CI)	RII (95% CI)
2016	56.94	78.40	49.78	17.78 (11.64, 23.93)	1.38 (1.29, 1.46)
2017	58.37	79.60	50.18	17.45 (11.64, 23.93)	1.36 (1.28, 1.44)
2018	60.09	80.55	50.60	17.59 (11.51, 23.67)	1.35 (1.27, 1.43)
2019	61.37	79.89	52.62	15.46 (9.25, 21.67)	1.29 (1.22, 1.37)
2020	62.50	79.63	52.81	13.69 (7.47, 19.91)	1.25 (1.18, 1.32)
2021	63.03	77.94	53.08	12.25 (5.86, 18.63)	1.22 (1.16, 1.29)

CI – confidence interval, RII – relative index of inequality, SII – slope index of inequality

### SC dimension

**Figure 4:**
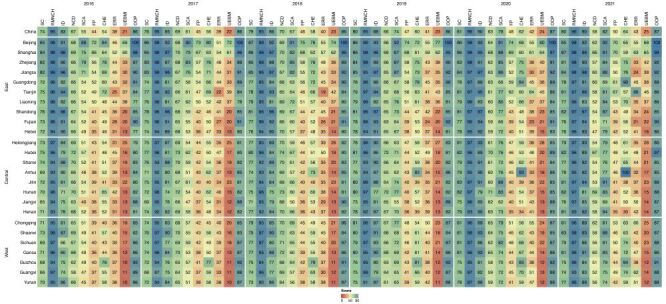
Scores of dimensions, domains, and indicators by province across time (2016–21). CHE – catastrophic health expenditure, ERR – effective reimbursement rate, FP – financial protection, ID – infectious disease, NCD – non-communicable disease, OOP – out-of-pocket payment, RMNCH – reproductive, maternal, newborn, and child health, SC – service coverage, SCA – service capacity and access, UEBMI – urban employee basic medical coverage,

An improvement in the SC dimension was observed in China, although a decline occurred in 2021. The index rose from 73.74 in 2016 to a climax of 80.57 in 2020 before slightly decreasing to 80.12 in 2021. SC exhibited a faster rate of progress at 1.34% (95% CI = 0.52, 2.15) compared to FP, which was 1.15% (95% CI = 1.04, 1.27). The RMNCH achieved near-universal coverage within the four domains with minimal change over time. ID and SCA have developed rapidly, whereas NCD has worsened since 2019, imposing a heavier burden on the country (**Figure 4**).

In alignment with the national characteristics, most provinces exhibited a combination of relatively high indicators for ID and RMNCH but lower scores for NCD. Most attained RMNCH scores exceeding 90 across the years, implying extensive health services coverage for women and children. In 2021, the lowest ID score has exceeded 80. Regarding temporal trends, a significant rise occurred in SCA across all provinces except Zhejiang, where GPs and hospital bed density dropped after 2020. GP density showed the most significant changes among all indicators, particularly in the Central and Western provinces. For example, in Shaanxi, the number of GPs per 10 000 residents has increased from 0.72 in 2016 to 3.35 in 2021. In contrast, all provinces experience a decline in NCD. The highest NCD value has not reached 70 in 2021, with Hebei recording the lowest at 46.92. This decline could be attributed to increased alcohol consumption and decreased participation in regular physical exercise. In Tianjin, the proportion of individuals engaging in frequent physical activity decreased from 37.6% in 2016 to 15.8% in 2021.

Owing to the varying pace of progress among the provinces, the results presented a narrowing inequality from 2016 to 2020, except in the NCD domain. The SII has halved from 12.68 (95% CI = 8.30, 17.06) in 2016 to 5.11 (95% CI = 1.73, 8.50) in 2020 but slightly recovered to 5.47 (95% CI = 1.77, 9.18) in 2021. Likewise, RII reduced from 1.19 (95% CI = 1.14, 1.24) to a minimum of 1.07 (95% CI = 1.03, 1.10) but increased in 2021 (Appendix S6 in the **Online Supplementary Document**). This trend could be attributed to the greater progress achieved in the provinces located in the Western and Central regions. Provinces in the Eastern region, which initially had relatively high scores, experienced slow progress, with some exhibiting reversed trends, as observed in Beijing and Zhejiang. However, the patterns of NCD were distinct. Provinces in the Western and Central regions had more modest reductions, whereas the Eastern provinces declined more, particularly in indicators measuring risky health behaviours. This expansive gap contributed to the widening difference in the SC dimension.

### FP dimension

The national index for the FP dimension increased from 43.96 in 2016 to 49.58 in 2021 (**Figure 4**). This increase indicated a reduction in the number of individuals vulnerable to financial hardship caused by health care expenses. The incidence of CHE contributed most to the index change. Using the 40% threshold, the proportion of households with CHE decreased from 13.53–10.83%.

Variations existed among provinces, as some have performed better in terms of financial protection than others. In 2021, the index ranged from 34.95–70.13, with Beijing having the highest value and Henan the lowest. The performance often corresponded to the levels achieved across individual indicators. Provinces in the Eastern region, with higher values, generally exhibited broader urban employee basic medical insurance (UEBMI) coverage and more generous inpatient reimbursements. Provinces in the Central region, with relatively low values, scored low across these indicators, particularly for UEBMI coverage. UEBMI coverage and OOP remained stable across most provinces, except for Hubei, where OOP increased. The incidence of CHE was the indicator of the most significant change. Most Central provinces, such as Anhui and Henan, experienced deterioration, whereas most Western provinces, such as Sichuan and Guizhou, improved.

Despite the existing variations, we observed a gradual decrease in provincial disparities. RII slightly increased from 1.61 (95% CI = 1.47, 1.77) to 1.63 (95% CI = 1.41, 1.69), followed by a jump to 1.40 (95% CI = 1.28, 1.54) in 2021 (Appendix S6 in the **Online Supplementary Document**). The faster rates of progress observed in the Western provinces were a possible cause for this trend (**Figure 2**). This trend was consistent with the changes in ERR and OOP payments. The only exception was the occurrence of CHE. Although the total fraction decreased, the difference increased over time. In 2021, the incidence in Henan (20.00%) was more than five times higher than in Guangdong (3.69%).

## DISCUSSION

To the best of our knowledge, this is one of the first studies in China to comprehensively assess UHC using a series of indicators encompassing SC and FP at the provincial level. Our findings indicated that most provinces in China have made commendable progress in achieving UHC targets, but the pace has slowed down. With nearly universal coverage of essential RMNCH services and rapid improvements in SCA, most provinces exhibited higher scores and faster increases in SC than in FP. In contrast, most Western provinces presented faster rates of progress owing to more significant increases in FP. Narrowing inequalities between provinces were observed across the overall index and its two dimensions. However, the exception occurred in the NCD domain, where most indicators experienced significant declines, and the differences continued to widen, particularly involving risky health behaviours.

Compared to prior studies, we extended the scope of tracer indicators for NCD prevention to include behavioural risk factors. Additionally, we expanded our measures to accurately reflect the financial burden experienced by the Chinese population. While rescaling indicators, imputing missing data, and determining weighting schemes, we consulted various global reports, relevant literature, and experts. This index demonstrated comparability with previous findings and exhibited a robust performance in various sensitivity tests, thereby verifying the reliability and validity of our methodology. Our SC score closely matched the findings of similar studies, with most indicating a national value exceeding 80 [6,8,9,11,28,29]. Although the FP score was comparably lower than in previous studies due to differences in indicator selection, the results regarding CHE incidence remained broadly consistent [15,27,44].

The national UHC index masked substantial variations across provinces. By disaggregating data, this study revealed that provinces with the most rapid progress, such as Tianjin, Chongqing, and Guangxi, were predominately located in the Western region, where socioeconomic levels were relatively low and resource allocations had not reached their full potential. These provinces demonstrated a more remarkable improvement in FP than in SC. By contrast, provinces in the Central region generally exhibited slower progress. The pace has decelerated in the Eastern region, with some provinces displaying an inverse trend. Factors within and outside the health system could have contributed to the variability in UHC progress across provinces. First, macroeconomic characteristics such as GDP growth were significantly associated with UHC performance [27,29]. Western provinces experienced a larger average annual percentage change in GDP per capita from 2016 to 2021 [45]. Second, policies have also been implemented to promote balanced regional development. Western provinces benefited particularly from initiatives such as the health poverty alleviation programme. This programme provided free preventive services and consolidated multi-tier health security systems, primarily in poverty-stricken areas of Western China, reducing OOP by 15% and CHE by 7.7% [18]. Moreover, the development of health infrastructure was crucial. Governments in the Western region demonstrated a heightened commitment to constructing an integrated care system by investing more in capacity building of primary health care facilities, including community and township health centres [19]. Finally, it also implied that achieving marginal improvements in high-standard regions may pose challenges, particularly in Eastern provinces with comparatively elevated scores.

Regarding SC, provinces generally performed highly, particularly in the RMNCH and ID domains. This trend was reflected in countries with similar sociodemographic settings, such as Thailand, Malaysia, and India [6,8]. Strong political will was a pivotal contributor to this circumstance [46]. Since 2009, the central government has offered stable financial investments for the national essential public health services programme, including free and universal RMNCH interventions [47,48]. Another crucial determinant was community consensus and multi-sectoral collaboration outside the health system. In 2019, over 700 civil society organisations dedicated to HIV/AIDS played pivotal roles in promoting the management of infectious diseases [49]. Additionally, the results indicated that GP density in the SCA domain emerged as the indicator of rapid growth in Central and Western China. China’s GP system was established in 2011, with pertinent policies concentrating on GP training and incentive mechanisms. The tilt of the national policy, together with the increase in government health expenditure, facilitated more rapid development in Central and Western provinces [50,51]. Nonetheless, it is necessary to note that those provinces continued to face a GP workforce shortage compared to the Eastern provinces and established policy goals. However, the increasing prevalence of NCD in China poses severe challenges [36]. The situation worsened as more individuals engaged in risky behaviours and inter-provincial disparities widened. This deterioration may be due to generally low levels of health literacy and increased exposure to prevalent unfavourable lifestyles. The pandemic of the coronavirus disease 2019 (COVID-19) has exacerbated this inequality [52].

Regarding FP, the results indicated that most provinces fell short of universal coverage. In 2021, Beijing scored the highest at 70.13, whereas Henan attained the lowest at 34.95. China’s FP performance also lagged behind that of countries at similar developmental stages, such as Thailand and Malaysia [53]. Thailand’s success in achieving robust financial protection through a comprehensive benefits package and implementing a tax-financed universal coverage scheme offers valuable insights [54]. Our findings revealed that CHE played a significant role in these shifts and worsened in more than half of the provinces. The first reason for this trend may stem from the overutilisation of tertiary health care services, reflecting a fragmented health care system that remained hospital-centric [55]. The adverse effects of the COVID-19 pandemic on the economy may also be a contributing factor [3,56]. Low economic activity increased unemployment rates and decreased household incomes, driving more families into financial hardship [57].

To further improve UHC levels and equity in China, we recommend that governments fully consider multiple factors, both within and outside the health system, when devising practical strategies. Equity can only be achieved by developing specific intervention programmes at the subnational or granular levels by identifying the targeted population [58]. Resources can be allocated to economically underdeveloped areas in the Central and Western regions to address the challenges in SCA and FP. Strategies for enhancing service capacity development, particularly for the GP workforce, involve improving educational inputs and redesigning incentive mechanisms. Internet plus health initiatives could empower service accessibility and availability [50]. Measures for financial coverage include reforming health insurance schemes to mitigate catastrophic health expenditures. Redesigning the medical assistance scheme to provide tiered and generous support to vulnerable beneficiaries is of particular concern [46]. In economically developed areas of Eastern China, developing prevention-oriented policies for NCD could decrease treatment costs and alleviate financial burdens [55]. Various concerted interventions can target risk factors and engage multiple sectors, including increasing tobacco and alcohol taxes, implementing physical activity programmes, investing in public leisure facilities, and adopting social media for health education promotion [26,52].

There are several limitations in our study. First, excluding six provinces may have overestimated the UHC process and underestimated equity. Except for Hainan, the remaining five provinces are all located in Western China, with relatively backward socioeconomic development levels and lower UHC index scores, as reported in relevant literature [29]. Future work should focus on sourcing more reliable data, particularly from economically advantaged areas, to enhance the monitoring of health inequality. Second, the selection of proxy indicators did not reflect effective service coverage. Some crucial indicators were also excluded due to data accessibility, suggesting that future work is required to acquire high-quality data from multiple sources. Third, more careful consideration should be given to the differences between provinces. Since the UHC emphasises structural and procedural elements of the health system, the assessment did not consider health outcomes. Consequently, a high level of UHC does not necessarily correlate with high-quality services or high responsiveness among the population. Moreover, more detailed studies should be conducted to forecast the UHC levels achieved by each province by 2030. Further investigations are necessary to explore the causality of policy implementation at UHC levels and examine the underlying mechanism.

## CONCLUSIONS

UHC is a critical element of the SDGs and the ‘Healthy China 2030’ initiative, leading China to prioritise its integration into health care policies and achieve significant advancements from 2016 to 2021. Provincial disparities narrowed across the 25 provinces, although the overall levels of UHC diverged. The worsening status and widening gap in the NCD domain provide important implications for identifying necessary interventions. These results highlight the urgency for less-developed regions to continuously enhance service capacity and financial protection, particularly by improving the GP workforce supply and mitigating catastrophic payments. Developed regions should prioritise addressing NCDs through effective and efficient interventions targeting key risk factors. Collectively, these crucial interventions facilitate the equitable realisation of UHC in China. This study provides valuable global insights into comprehensively monitoring UHC, uncovering subnational disparities, and formulating context-specific policy initiatives.

## Additional material


Online Supplementary Document
