# Supplementary material for: Provincial inequality of China’s progress towards universal health coverage: An empirical analysis in 2016–21
Source: J Glob Health. 2024 Jun 28;14:04122. doi: 10.7189/jogh.14.04122 (PMC11211969; doi:10.7189/jogh.14.04122)
Supplement: Online Supplementary Document [file jogh-14-04122-s001.pdf]

Supplementary Materials

Appendix S1: Principles for our choice of indicators 2

Appendix S2: Introduction of sources and sampling of CFPS 3

Appendix S3: Detailed definition, sources and transformation of selected indicators 4

Appendix S4: Average annual changes in UHC index by province 8

Appendix S5: Trends in SC score and FP score by province 9

Appendix S6: SII and RII for two separate dimensions 10

Appendix S7: Sensitivity analysis results 11

Appendix S1: Principles for our choice of indicators

Four principles underpinned our choice of tracer indicators for the overall UHC index. First, the principle of scientization. We based our monitoring framework on the one proposed by WHO and World Bank in 2017, which has been continuously used in global monitoring reports released by WHO annually, and supplemented it by reviewing relevant literatures. In order to evaluate UHC process in China, we tailored it to China's specific context through expert interviews. Second, the principle of systematization. The selected indicators should cover a wide range of supply-side factors (doctors, nurses, etc.), demand-side factors (children, pregnant women, the elderly, etc.), and service categories (preventive services, treatment services, etc.) within the health system so as to conduct a comprehensive and objective evaluation. Third, the principle of purposefulness. The selected indicators should reflect China's priorities in the process of reaching UHC. Also, they should have a visionary target in the official policy documents from different organizations such as WHO and the Health Commission in China. Fourth, the principle of accessibility. It means that the data should be as accessible as possible, with raw data covering the selected years of the study.

Appendix S2: Introduction of sources and sampling of CFPS

China Family Panel Studies (CFPS) was designed by the Peking University research team from 2010. It is China's first large-scale academically-oriented longitudinal survey project. By collecting data at three levels (i.e., individual, family, community), the project aims to provide multi-dimensional, multi-level and high-quality data for understanding rapid-changing social phenomena in China in various domains, including economy, population, education, and health. The baseline survey was implemented in 2010 and five waves of full sample follow-up surveys have already been conducted every two years in 2012, 2014, 2016, 2018, and 2020. The CFPS baseline sample covers 25 provinces/municipalities/autonomous regions in China (excluding Hong Kong, Macao, Taiwan, Xinjiang, Tibet, Qinghai, Inner Mongolia, Ningxia and Hainan), representing over 95% of the Chinese population. CFPS implemented Probability-Proportional-to-Size Sampling (PPS) with implicit stratification. Administrative units and socioeconomic status (SES) were used as the main stratification variables. Within the administrative unit, local GDP per capita was used as the ordering index for SES. If the GDP per capita in the administrative unit is not available, the proportion of non-agricultural population or population density is used. All the sub-sampling frames of CFPS were obtained through three stages: the Primary Sampling Unit (PSU) consisted of administrative districts/counties, the Second-stage Sampling Unit (SSU) consisted of administrative villages/ neighborhood communities, and the third-stage (Ultimate) Sampling Unit (TSU) consisted of households determined by systematic sampling. The baseline survey ended with interviewing a total of 14,960 households and 42,590 individuals. In 2018, a total of 15,051 households completed the family roster questionnaires and nearly 44,000 individuals completed the interviews. At the household level, the CFPS2018 had a response rate of 69.3% and cross-wave retention rate of 86.6%. in 2020, the sample exceeded 22,000 households and 66,000 individuals, although the response rate and the retention rate slightly dropped as COVID-19 restricted face-to-face interviews.

References

[1] Xie Y, Zhang X, Tu P, et al. China Family Panel Studies User's Manual (3rd edition). Institute of Social Science Survey, Peking University, July 2017

[2] Wu Q, Dai L, Zhen Q, Gu L, Wang Y. User Guide for China Family Panel Studies 2018. Institute of Social Science Survey, Peking University, August 2021

[3] Ta Y, Zhu Y, Fu H. Trends in access to health services, financial protection and satisfaction between 2010 and 2016: Has China achieved the goals of its health system reform? Soc Sci Med 2020; 245: 112715.

Appendix S3: Detailed definition, sources and transformation of selected indicators

Indicator	Definition	Data source	Transformation formula	Accessible year	Rational
**Service Coverage (SC): Reproductive, Maternal, Newborn and Child Health (RMNCH)**					
Maternal systematic management	The ratio of the number of puerperae in maternal systematic management to the number of live births. Maternal systematic management includes early pregnancy test, at least five antenatal visits, new midwifery and postpartum visit between pregnancy and 28 days after delivery.	China Health Statistical Yearbook	Score = Maternal systematic management * 100	2016–2021	[1-4,6,9-11]
Skilled birth attendance	The share of live births delivered in institutions qualified to assist in childbirth.	China Health Statistical Yearbook	Score = Skilled birth attendance * 100	2016–2021	[2-4,6,11]
Full immunization	The share of children vaccinated for 22 National Immunization Program (NIP) doses.*	Relevant literatures	Score = Full immunization *100	2016–2021	[1,4,9,11]
Child systematic management (age 0-3)	The share of children (age 0-3) in child systematic management. Systematic management includes growth monitoring or 4:2:1 (urban) or 3:2:1 (rural) physical examination checks (height and weight) by age.	China Health Statistical Yearbook	Score = Child systematic management (age 0-3) * 100	2016–2021	[1,9-10] (proxy)
**Service Coverage (SC): Infectious Disease (ID)**					
Incidence of Pulmonary Tuberculosis (TB)	The incidence rate of Pulmonary Tuberculosis per 100k in an area in a given year.	China Health Statistical Yearbook	Score = (378.00- Incidence of TB) / (378.00-3.59) * 100	2016–2021	[1-2,9-10] (proxy)
Incidence of HIV/AIDS	The incidence rate of Acquired Immune Deficiency Syndrome (AIDS) in an area in a given year.	China Health Statistical Yearbook	Score = (526.23- Incidence of HIV/AIDS) / (526.23-0.30) * 100	2016–2021	[1,9-10] (proxy)
Improved water source	The share of administrative villages whose water is taken centrally from the water source, purified and disinfected as necessary, and then transported to users or centralized water supply points through the distribution network.	China Social Statistical Yearbook	Score = Improved water source * 100	2016, 2017, 2020, 2021	[1-4.9]
**Service Coverage (SC): Non-communicable Disease (NCD)**					

Prevalence of hypertension	The proportion of people with hypertension in the population surveyed.	China Hypertension Survey (CHS)、China Chronic Disease and Risk Factor Surveillance (CCDRFS)	Score = (1 - Prevalence of hypertension) * 100	2015, 2018	[1-2,7-8] (proxy)
Prevalence of diabetes	The proportion of people with diabetes in the population surveyed.	Global Burden of Diseases (GBD) Study 2019, China Chronic Disease and Risk Factor Surveillance (CCDRFS)	Score = (1 - Prevalence of diabetes) * 100	2016–2021	[1-2,7-8,10] (proxy)
Tobacco use	The percent of respondents (aged 15 years or older) who have not smoked cigarettes in the past month.	China Family Panel Study (CFPS)	Score = (1 - Tobacco use) * 100	2016, 2018, 2020	[1,3-4,7-9]
Alcohol consumption	The percent of respondents (aged 15 years or older) who have drunk alcohol at least 3 times a week in the past month.	China Family Panel Study (CFPS)	Score = (1 - Alcohol consumption) * 100	2016, 2018, 2020	[7-8]
Frequent physical exercise	The percent of respondents (aged 15 years or older) who have participated in physical exercise equal to or greater than three times in the past week.	China Family Panel Study (CFPS)	Score = Frequent physical exercise * 100	2016, 2018, 2020	[7-8]
**Service Coverage (SC): Service Capacity Access (Capacite)**					
Practicing (Assistant) Physician density	The number of Practicing (Assistant) Physicians per 1,000 residents, which can be further divided into four types, clinical, traditional Chinese medicine (TCM), dentistry and public health.	China Health Statistical Yearbook	Score = Practicing (Assistant) Physician density / 3.2 * 100	2016–2021	[1,5,9]
General Practitioner (GP) density	The number of General Practitioners (GP) per 10,000 residents.	China Health Statistical Yearbook	Score = General Practitioner (GP) density / 3.93 * 100	2016–2021	[5]
Registered nurse density	The number of Registered nurses per 1,000 residents.	China Health Statistical Yearbook	Score = Registered nurse density / 4.7 * 100	2016–2021	[5,9]
Hospital bed density	The number of beds in medical institutions per 1,000 residents.	China Health Statistical Yearbook	Score = Hospital bed density / 8.34 * 100	2016–2021	[1,9]
**Financial Protection (FP)**					
Incidence of catastrophic health expenditure (CHE)	The proportion of households whose medical expenditure is at least 40% of total expenditure net of food consumption.	China Family Panel Study (CFPS)	Score = (28%- Incidence of CHE) / (28%-1%)*100	2016, 2018, 2020	[1,3-6,10-11]

UEBMI coverage	The proportion of people who participate in Urban Employee Basic Medical Insurance (UEBMI).	China Statistical Yearbook, Health Statistical Yearbook, and China Medical Insurance Statistical Yearbook	Score = UEBMI coverage * 100	2016-2021	[4-6]
Effective reimbursement rate	The ratio of the total amount reimbursed or to be reimbursed over total medical cost (including both inpatient and outpatient services) for those who have been hospitalized within one year prior to the survey.	China Family Panel Study (CFPS)	Score = Effective reimbursement rate * 100	2016, 2018, 2020	[5-6]
Out-of-pocket payment	The ratio of out-of-pocket payment to total health expenditure.	China Health Statistical Yearbook	Score = (100 - Out-of-pocket payment) / (100-17.15) * 100	2016-2021	[5]

*22 NIP doses include Bacilli Calmette-Guérin vaccine (BCG), Hepatitis B vaccine (HepB), Poliomyelitis vaccine (PV), Diphtheria, tetanus and acellular pertussis combined vaccine (DTaP), Diphtheria and tetanus combined vaccine (DT), Measles containing vaccine (MCV), Hepatitis A vaccine (HepA), Japanese encephalitis attenuated live vaccine (JEV-L), Group A meningococcal polysaccharide vaccine (MPV-A), and Group A and C meningococcal polysaccharide vaccine (MPV-AC).

References

[1] World Health Organization, World Bank. Tracking Universal Health Coverage: 2021 Global Monitoring Report. World Health Organization: 2021.
[2] Feng XL, Zhang Y, Hu X, Ronsmans C. Tracking progress towards universal health coverage for essential health services in China, 2008-2018. BMJ Glob Health. 2022;7.
[3] Li Y, Zhang C, Zhan P, Fu H, Yip W. Trends and projections of universal health coverage indicators in China, 1993-2030: An analysis of data from four nationwide household surveys. Lancet Reg Health West Pac. 2023;31:100646.
[4] Zhou Y, Li C, Wang M, Xu S, Wang L, Hu J, et al. Universal health coverage in China: a serial national cross-sectional study of surveys from 2003 to 2018. Lancet Public Health. 2022;7:e1051-e63.
[5] Liu X, Wang Z, Zhang H, Meng Q. Measuring and evaluating progress towards Universal Health Coverage in China. J Glob Health. 2021;11:08005.
[6] Meng Q, Xu L, Zhang Y, Qian J, Cai M, Xin Y, et al. Trends in access to health services and financial protection in China between 2003 and 2011: a cross-sectional study. Lancet. 2012;379(9818): 805-14.
[7] Zhou Y, Wu Q, Li C, Meng P, Ding L. Inequalities in non-communicable disease management in China and progress toward universal health coverage: an analysis of nationwide household survey data from 2004 to 2018. Lancet Reg Health West Pac. 2023;44:100989.

[8] Nguyen PT, Gilmour S, Le PM, Nguyen HL, Dao TMA, Tran BQ, et al. Trends in, projections of, and inequalities in non-communicable disease management indicators in Vietnam 2010-2030 and progress toward universal health coverage: A Bayesian analysis at national and sub-national levels. EClinicalMedicine. 2022;51:101550.
[9] Hogan DR, Stevens GA, Hosseinpoor AR, Boerma T. Monitoring universal health coverage within the Sustainable Development Goals: development and baseline data for an index of essential health services. Lancet Glob Health. 2018;6:e152-e68.
[10] GBD 2019 Universal Health Coverage Collaborators. Measuring universal health coverage based on an index of effective coverage of health services in 204 countries and territories, 1990-2019: a systematic analysis for the Global Burden of Disease Study 2019. Lancet. 2020;396:1250-84.
[11] Wagstaff A, Neelsen S. A comprehensive assessment of universal health coverage in 111 countries: a retrospective observational study. Lancet Glob Health. 2020;8:e39-e49.

Appendix S4: Average annual changes in UHC index by province

		UHC (95% CI)	SC (95% CI)	FP (95% CI)
Overall	China	1.262*** [0.994,1.530]	1.338* [0.522,2.153]	1.153*** [1.038,1.269]
East	Beijing	-0.203 [-1.082,0.675]	-0.183 [-0.609,0.243]	-0.217 [-1.520,1.086]
	Shanghai	0.611*** [0.519,0.702]	0.496** [0.221,0.771]	0.695*** [0.549,0.841]
	Zhejiang	-0.198 [-1.103,0.707]	-0.297 [-1.262,0.668]	-0.125 [-0.965,0.715]
	Jiangsu	-0.304 [-1.100,0.492]	0.444 [-0.151,1.039]	-0.763 [-1.645,0.119]
	Guangdong	1.468*** [1.127,1.809]	1.118* [0.435,1.802]	1.703*** [1.594,1.812]
	Tianjin	2.985** [1.328,4.643]	1.296*** [0.880,1.712]	4.115* [1.306,6.923]
	Liaoning	0.759* [0.277,1.240]	0.820 [-0.553,2.193]	0.693*** [0.524,0.861]
	Shandong	0.715* [0.008,1.423]	1.120* [0.330,1.910]	0.447 [-0.172,1.066]
	Fujian	0.132 [-0.243,0.507]	0.523 [-0.389,1.436]	-0.084 [-0.210,0.042]
	Hebei	1.276*** [0.945,1.607]	0.872 [-0.309,2.053]	1.378*** [1.234,1.521]
Central	Heilongjiang	1.161** [0.627,1.695]	1.370* [0.539,2.202]	0.976** [0.510,1.441]
	Hubei	1.382*** [1.159,1.606]	1.428** [0.727,2.130]	1.272*** [1.031,1.512]
	Shanxi	1.082*** [0.893,1.272]	0.661* [0.044,1.279]	1.264** [0.851,1.676]
	Anhui	1.779*** [1.392,2.167]	1.951** [1.082,2.821]	1.576*** [1.485,1.668]
	Jilin	0.881*** [0.630,1.132]	2.044** [1.234,2.854]	0.210 [-0.031,0.451]
	Hunan	0.823** [0.536,1.110]	2.589** [1.676,3.501]	-0.171 [-0.409,0.068]
	Jiangxi	1.360** [0.856,1.865]	2.144*** [1.696,2.593]	0.833 [-0.084,1.751]
	Henan	0.481* [0.118,0.845]	1.814** [1.071,2.558]	-0.190* [-0.342,-0.038]
West	Chongqing	2.632*** [2.086,3.177]	2.078* [0.739,3.418]	2.833*** [2.768,2.899]
	Shaanxi	1.330** [0.872,1.788]	1.800** [1.169,2.430]	0.983** [0.643,1.323]
	Sichuan	2.016** [1.361,2.670]	1.930* [0.483,3.376]	1.963*** [1.815,2.112]
	Gansu	2.119*** [1.933,2.305]	2.096** [1.349,2.844]	1.976*** [1.805,2.147]
	Guizhou	0.979** [0.442,1.517]	1.351* [0.082,2.621]	0.707*** [0.560,0.853]
	Guangxi	2.261*** [1.692,2.830]	1.831* [0.367,3.296]	2.401** [1.030,3.772]
	Yunan	1.289*** [0.923,1.655]	1.527** [0.978,2.077]	1.070** [0.658,1.481]

UHC – Universal Health Coverage, SC – Service coverage, FP–Financial protection

95% confidence intervals in brackets

* $P < 0.05$, ** $P < 0.01$, *** $P < 0.001$

Appendix S5: Trends in SC score and FP score by province

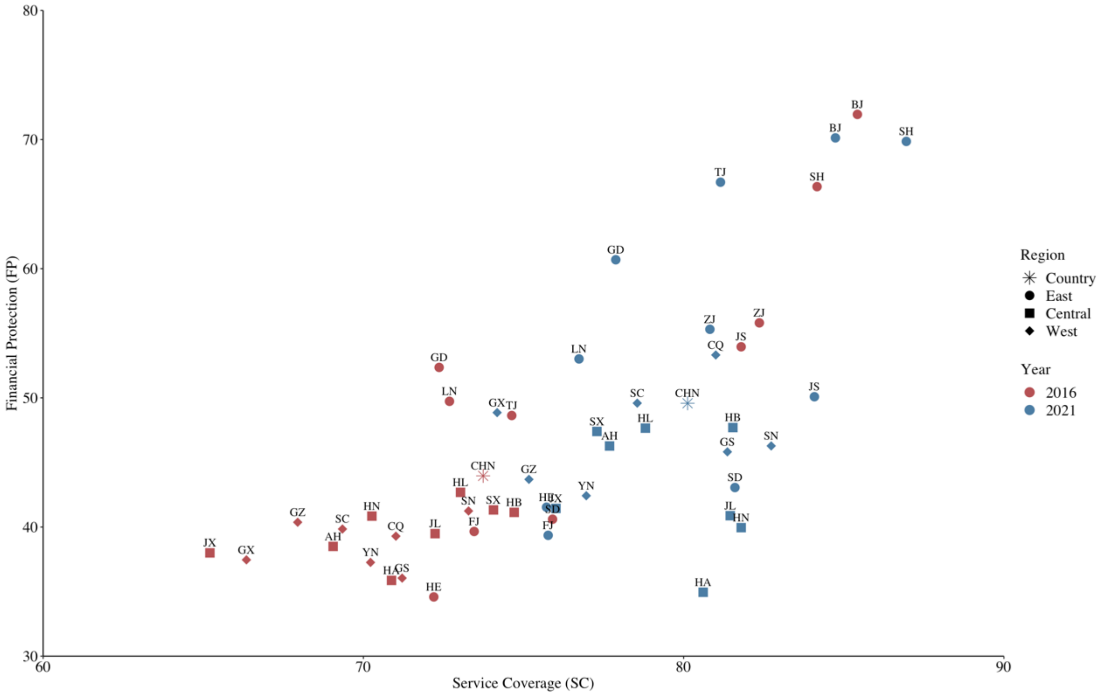

Figure 1. Trends in SC score and FP score by province between 2016 and 2021.

CHN–China; BJ–Beijing; TJ–Tianjin; HE–Hebei; SX–Shanxi; LN–Liaoning; JL–Jilin; HL–Heilongjiang; SH–Shanghai; JS–Jiangsu; ZJ–Zhejiang; AH–Anhui; FJ–Fujian; JX–Jiangxi; SD–Shandong; HA–Henan; HB–Hubei; HN–Hunan; GD–Guangdong; GX–Guangxi; CQ–Chongqing; SC–Sichuan; GZ–Guizhou; YN–Yunnan; SN–Shaanxi; GS–Gansu.

Appendix S6: SII and RII for two separate dimensions

	National Score	Highest Score by province	Lowest Score by province	SII (95% CI)	RII (95% CI)
Service Coverage dimension					
2016	73.74	85.43	65.21	12.68 (8.30, 17.06)	1.19 (1.14, 1.24)
2017	76.04	86.27	68.21	11.15 (7.14, 15.17)	1.16 (1.12, 1.20)
2018	78.69	86.65	70.72	9.89 (6.41, 13.37)	1.14 (1.10, 1.17)
2019	79.99	86.49	72.53	7.40 (3.91, 10.89)	1.10 (1.06, 1.13)
2020	80.57	86.29	75.02	5.11 (1.73, 8.50)	1.07 (1.03, 1.10)
2021	80.12	86.95	74.18	5.47 (1.77, 9.18)	1.07 (1.04, 1.11)
Financial protection dimension					
2016	43.96	71.95	34.59	20.33 (12.11, 28.55)	1.61 (1.47, 1.77)
2017	44.80	73.45	35.78	20.68 (12.33, 29.03)	1.61 (1.47, 1.76)
2018	45.88	74.87	36.05	21.61 (12.99, 30.22)	1.63 (1.48, 1.78)
2019	47.09	74.48	35.54	19.84 (10.79, 28.89)	1.55 (1.41, 1.69)
2020	48.48	74.07	35.42	18.52 (9.12, 27.92)	1.49 (1.36, 1.63)
2021	49.58	70.13	34.95	16.07 (5.96, 26.18)	1.40 (1.28, 1.54)

SII – slope index of inequality, RII – relative index of inequality, CI – confidence interval

Appendix S7: Sensitivity analysis results

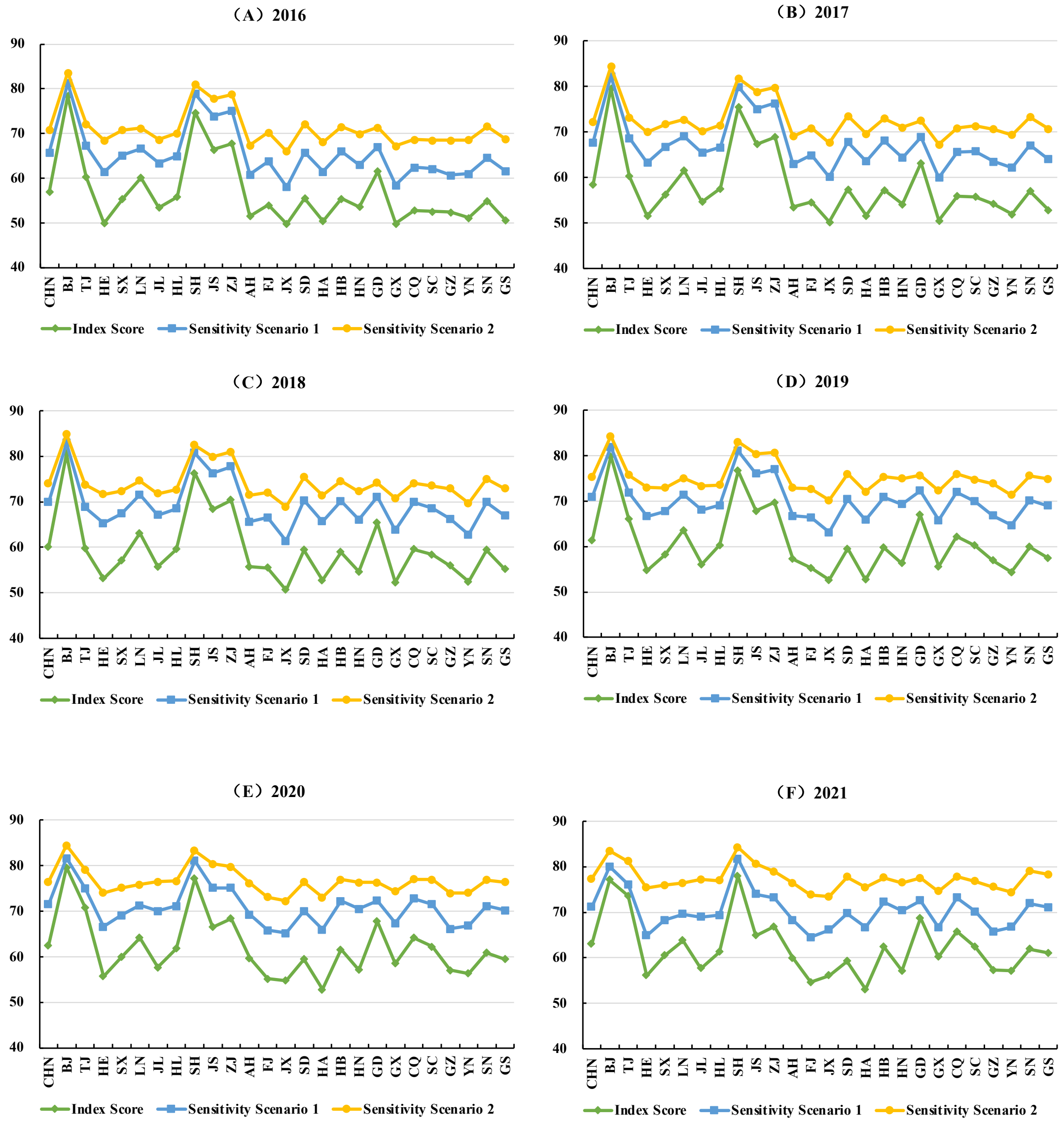

Figure1. Sensitivity analysis results (Scenario 1 and Scenario 2).

Index Score: original index; Scenario 1: genomic mean of each indicator; Scenario 2: arithmetic mean of each indicator.

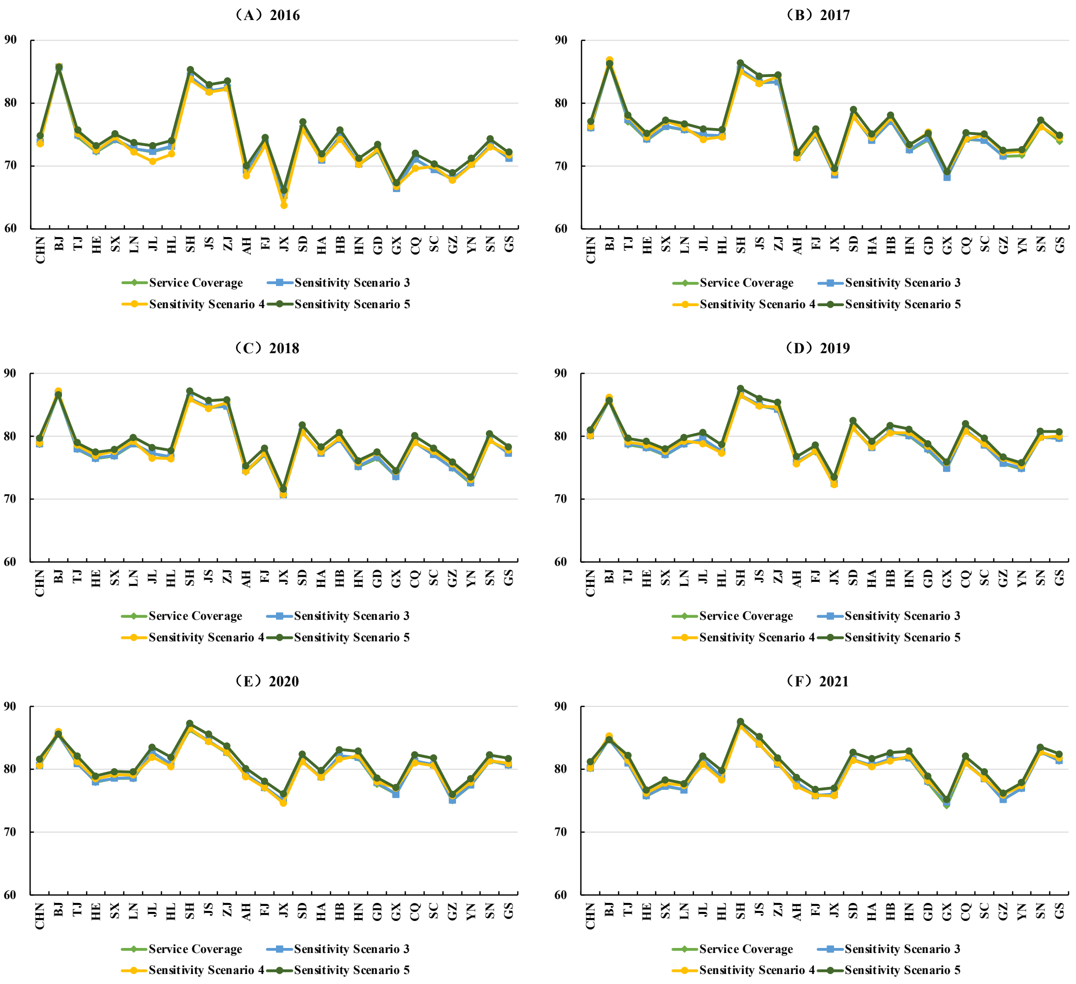

Figure 2. Sensitivity analysis results (Scenario 3, Scenario 4, and Scenario 5).

Service Coverage: original index; Scenario 3: replacing Child systematic management (age 0-3) with Child health management (age 0-7); Child health management (age 0-7): The share of children (age 0-7) in child health management. Health management includes physical examination checks (height and weight) during the year; Scenario 4: replacing Improved water source with Rural water sanitation coverage; Scenario 5: replacing Registered nurse density with scores using 3.8 per 1,000 residents as the target value.

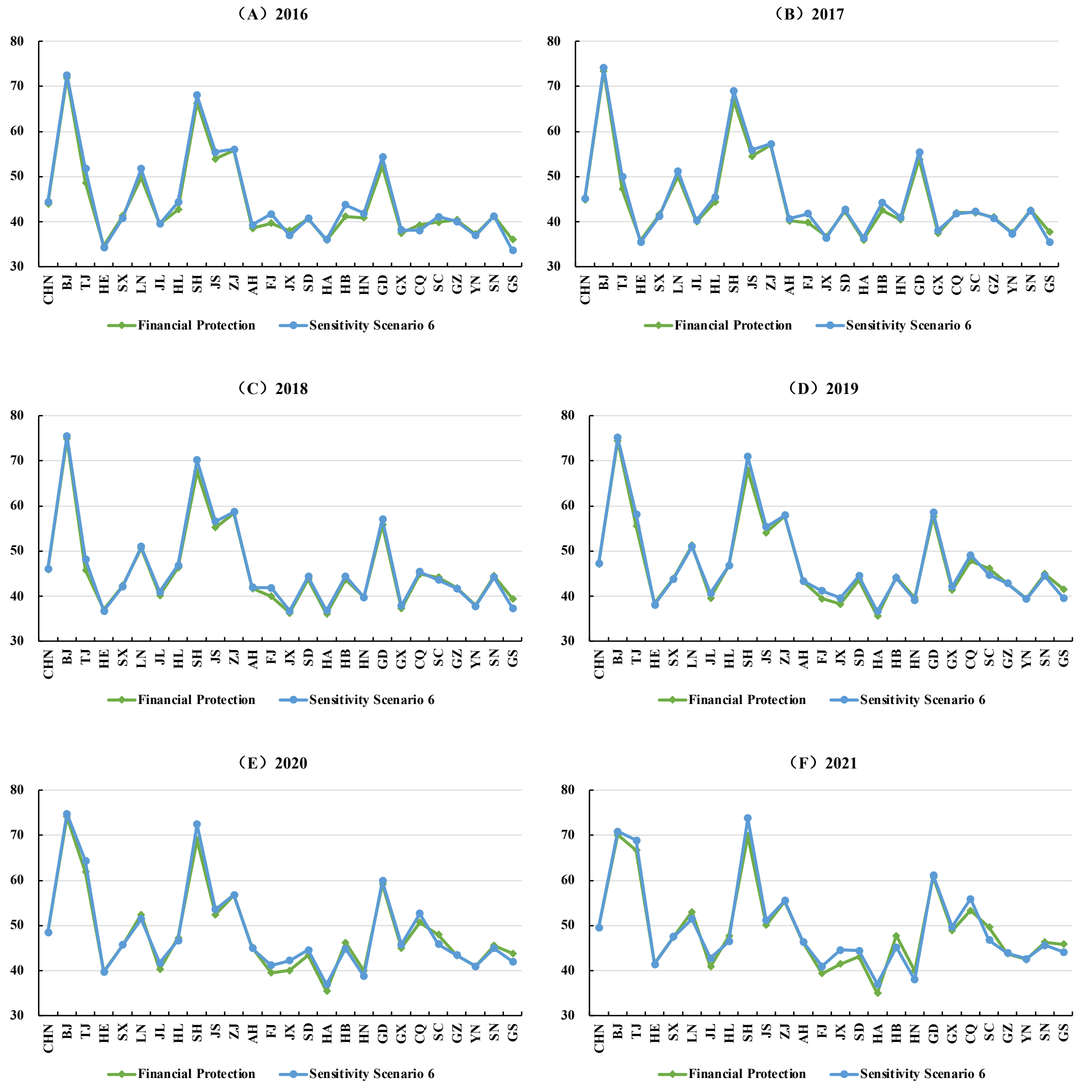

Figure 3. Sensitivity analysis results (Scenario 6).

Financial Protection: original index; Scenario 6: replacing Incidence of catastrophic health expenditure (CHE) (40% threshold) with Incidence of catastrophic health expenditure (CHE) (25% threshold); Incidence of catastrophic health expenditure (CHE) (25% threshold): The proportion of households whose medical expenditure is at least 25% of total consumption.
